# Proteomics reveal serum biomarkers for early diagnosis in premature ovarian insufficiency

**DOI:** 10.3389/fendo.2026.1917911

**Published:** 2026-09-17

**Authors:** Zheng Su, Penghui Feng, Siqi Sun, Jiaze Li, Yue Hou, Yueping Zeng, Xiaomin Hu, Qi Yu, Min Luo

**Affiliations:** 1 State Key Laboratory of Complex Severe and Rare Diseases, Peking Union Medical College Hospital, Chinese Academy of Medical Sciences & Peking Union Medical College, Beijing, China; 2 Department of Dermatology, Peking Union Medical College Hospital, Chinese Academy of Medical Sciences and Peking Union Medical College, National Clinical Research Center for Dermatologic and Immunologic Diseases, Beijing, China; 3 National Clinical Research Center for Women’s Health and Obstetric and Gynecologic Diseases, Department of Obstetrics and Gynecology, Peking Union Medical College Hospital, Chinese Academy of Medical Sciences & Peking Union Medical College, Beijing, China

**Keywords:** diagnostic biomarker, inflammatory dysregulation, label-free proteomics, premature ovarian insufficiency, S100A8/S100A9

## Abstract

**Background:**

Premature ovarian insufficiency (POI) leads to infertility with unclear mechanisms and scarce early diagnostic biomarkers. This study explored serum protein profiles to uncover POI molecular mechanisms and candidate biomarkers.

**Methods:**

Label-free serum proteomics was performed using serum samples from 20 POI patients, 20 healthy controls (HC) and 20 menopausal women (MP) to screen differentially expressed proteins (DEPs). Bioinformatic analyses including functional enrichment and correlation analysis were carried out. Candidate markers were evaluated by ROC analysis and validated by ELISA in an independent cohort of 88 participants.

**Results:**

Three groups had distinct proteomic profiles. We identified 54 DEPs between POI and HC, as well as 30 DEPs between POI and MP. Functional enrichment uncovered dysregulation in cytoskeleton organization and autoimmune response in POI. Five biomarkers (S100A8, S100A9, TUBA1A, TPM4, RAP1B) exhibited stable diagnostic performance across cohorts. The combined signature of S100A8 and S100A9 yielded reliable discriminatory power, and independent ELISA testing further validated their elevated serum levels and satisfactory diagnostic value for POI.

**Conclusions:**

POI is characterized by abnormal cytoskeleton homeostasis and inflammatory dysregulation. Five proteins, especially S100A8/S100A9, represent potential serum protein biomarkers for early POI diagnosis.

## 1 Introduction

Premature ovarian insufficiency (POI) is defined as a woman achieves menopause before the age of 40, also known as premature menopause or primary ovarian insufficiency (1). POI affects approximately 1-3.5% of females worldwide, resulting in the cessation of the menstrual cycle and infertility (1, 2). The underlying mechanisms causing POI are not comprehensively understood, although genetic mutations, environmental factors, infections, and autoimmunity have been shown to contribute to POI development (3). Presently, the diagnosis of POI is based on clinical presentation and the elevated serum levels of follicle-stimulating hormone (FSH) (1). However, the progression of POI is gradual, and reliable biomarkers for early diagnosis or prognostication during initial disease stages remain elusive. Several factors have been used to predict POI, such as age, anti-Mullerian hormone (AMH), estradiol (E_2_), inhibin B and FSH serum levels, antral follicle counts, menstrual cycle length, and some genetic markers (4). Although these factors can provide some insights, more specific and sensitive biomarkers are needed for early-stage diagnosis of POI.

Currently, genomics and transcriptomics analysis have been used to explore biomarkers of POI. Many studies on POI-related genes are focused on DNA repair, meiosis, mitochondrial functions, and immune infiltration (5–8). Although numerous studies have investigated POI, reliable protein biomarkers specific for POI diagnosis have not been established. Proteomic analysis involves the comprehensive identification and quantification of proteins within biological samples and serves as an indispensable tool for discovering biomarkers across diverse diseases. This study, therefore, employed quantitative proteomics of POI patient serum to identify potential diagnostic biomarkers and further verify their correlations with clinical indicators.

## 2 Methods

### 2.1 Study samples

In this study, a total of 20 healthy control (HC) individuals, 20 natural menopausal (MP) individuals, and 20 POI patients were recruited from the Gynecology Department of Peking Union Medical College Hospital, China. Prior to analysis, serum samples were collected from all participants in accordance with hospital standard operating procedures. The inclusion of women with POI requires the presence of oligo/amenorrhea for at least 4 months in women below the age of 40, and with an increased FSH serum concentration of > 40 IU/L detected on at least two separate occasions > 4 weeks apart. The inclusion of MP groups requires that the woman has no spontaneous menstruation for 12 consecutive months after the age of 40, excluding pregnancy and pathological factors. On the contrary, the healthy control group refers to people with regular menstruation before the age of 40. The participants were not under any medication before their blood samples were collected. The blood hormone levels of luteinizing hormone (LH), FSH, E_2_, Testosterone, and AMH were subsequently tested using standardized assays. Hormone concentrations below the assay limit of detection (LOD) were substituted with LOD/√2 for subsequent correlation analyses. The study protocol was approved by the Ethics Committee of Peking Union Medical College Hospital (approval number: K3080), and written informed consent was obtained from all participants.

### 2.2 Pretreatment of serum samples

The pretreatment steps for serum samples are as follows: Take a 30 μL serum sample and add 240 μL of methanol. Then, place it at -80 °C for overnight precipitation. Subsequently, centrifuge the sample and carefully collect the protein layer. Resuspend the protein layer in methanol, centrifuge again, air-dry it, and perform ultrasonic treatment before measuring the protein concentration. Next, disulfide bonds were reduced by incubating the protein sample with 10 mM dithiothreitol (DTT) at 56 °C for 45 min, followed by cysteine alkylation with 20 mM iodoacetamide (IAA) in the dark at room temperature for 30 min to ensure the stability of the protein structure. After that, sequencing-grade trypsin (Promega, USA) was added at an enzyme-to-substrate ratio of 1:50 (w/w), and digestion was performed at 37 °C for 16 h. Once the enzymatic digestion was completed, the pH was adjusted to <3 by adding trifluoroacetic acid (TFA) and the resulting peptide mixture was desalted using C18 ZipTips (Millipore, USA) following the manufacturer’s instructions. Finally, elute and quantify the treated sample, take an appropriate amount for proteomic analysis, and perform drying treatment.

### 2.3 Proteome screening and identification

LC-MS/MS analysis was performed on an instrument where an Orbitrap Fusion Tribrid mass spectrometer (Thermo Fisher Scientific) was coupled online with a nanoflow LC system (EASY-nLC 1200, Thermo Fisher Scientific). The polypeptide mixture was resuspended and injected into a specific trapping column for sample introduction. After ionization of the peptides, full MS scan data were acquired in data-dependent acquisition (DDA) mode. The obtained MS/MS spectra were processed using MaxQuant (v2.8.1.0) with its built-in Andromeda search engine and searched against the UniProtKB human proteome database. During the search process, parameters such as the mass tolerance for precursors and product ions, the allowed number of missed cleavage sites, and dynamic modifications were set. A 1% false discovery rate (FDR) was applied at the peptide level for screening.

Stringent contamination control was implemented during database searching in MaxQuant. Peptides were restricted to a length range of 7–25 amino acids to reduce false positive matches to exogenous contaminants. All identified proteins were annotated against a contaminant list including keratin, trypsin, hemoglobin, albumin, IgG, casein, lysozyme and BSA; proteins with low FDR or low confidence were flagged for exclusion. The number of proteins identified in each sample is summarized in **Supplementary Table 1**.

### 2.4 Protein quantification and differential analysis

Protein quantification was performed using a label-free quantification method. In the Proteome Discoverer software, the intensity of the extracted ion current (XIC) was used to relatively quantify the proteins. By integrating the peak areas of the same peptides in different samples, the relative abundance of each protein in each sample was calculated. For missing values in the data, a K-nearest neighbors (KNN) algorithm was used for imputation. The K value was set to 50% of the total number of samples. The principle of this algorithm is to find the K nearest neighbor samples to the sample with missing values in the dataset and estimate the missing values by weighted averaging based on the protein abundance data of these neighbor samples. After imputing the missing values, the data were log10 transformed. For differential statistics, the limma package in R was adopted; linear models incorporating experimental groups and age as a covariate were constructed for each protein, followed by empirical Bayes moderation and Benjamini–Hochberg multiple testing correction. Proteins with |log_2_FC|≥1 and adjusted P-value ≤ 0.05 were considered differentially expressed proteins. To exclude age confounding, an independent limma correlation model was built to assess the correlation between each protein and chronological age.

### 2.5 Protein function analysis

The screened proteins were subjected to functional enrichment analysis. The clusterProfiler package in the R language was used to conduct gene ontology (GO) analysis and Kyoto Encyclopedia of Genes and Genomes (KEGG) pathway enrichment analysis. First, the gene names of the differentially expressed proteins were converted into a format suitable for analysis by the R package. The enrichGO function in the clusterProfiler package was used to perform GO enrichment analysis on the differentially expressed proteins. The OrgDb parameter was set to “org.Hs.eg.db” to specify the human genome database, and the ont parameter was set to “ALL” to comprehensively explore the main biological functions of the differentially expressed proteins from three aspects: biological process, cellular component, and molecular function. Meanwhile, the enrichKEGG function was used for KEGG pathway enrichment analysis, and the organism parameter was set to “hsa” to represent the human species, aiming to identify the metabolic pathways and signal transduction pathways in which the differentially expressed proteins were significantly enriched.

### 2.6 Protein-protein interaction network analysis

The online tool of the STRING database was used to retrieve the interaction relationships between the target proteins with a confidence threshold of 0.7, and a protein-protein interaction network was constructed. The interaction network file was downloaded from the STRING database and imported into the Cytoscape software for further analysis. In Cytoscape, the MCODE (Molecular Complex Detection) module was used to identify closely connected subnetworks in the complex protein-protein interaction network, thereby analyzing the core modules of the network. At the same time, the degree and the number of edges of each protein were counted. Proteins were ranked according to their degree and node size, and those with higher degree and node size values were selected as key proteins.

### 2.7 Differential statistics and visualization

Differential expression analysis was performed using the limma package in R, fitting a linear model for each protein with a design matrix incorporating experimental groups and age as a confounding covariate. Pairwise contrasts of interest were defined, and empirical Bayes moderation was applied via the eBayes function to moderate protein-wise variances and generate moderated t-statistics and p-values. To correct for multiple testing, p-values were adjusted using the Benjamini–Hochberg method, and proteins with |log_2_FC|≥1 and adjusted p-value < 0.05 were defined as significantly differentially abundant. For simple two-group comparisons of clinical indicators, Student’s t-test was adopted for normally distributed data and Wilcoxon rank-sum test for non-normal data; P < 0.05 was defined as the statistical threshold. All analyses were run in RStudio. The mixOmics package was utilized for Principal Component Analysis (PCA) and Partial Least Squares Discriminant Analysis (PLS-DA) dimensionality reduction, while ggplot2 was used to generate volcano plots, heatmaps, bar charts and box plots.

### 2.8 ELISA validation

Following the proteomic screening, serum concentrations of the selected proteins were quantified using specific ELISA kits according to the manufacturer’s instructions. Briefly, diluted serum samples from all participants (POI patients, MP individuals, and HC individuals) were added to pre-coated wells. After incubation and washing steps, the absorbance was measured at the specified wavelength. Protein concentrations were determined by interpolating from the standard curve run in parallel with each assay. All samples were tested in duplicate.

## 3 Results

### 3.1 Baseline characterization of all participants

The baseline characteristics of the three study groups were summarized in **Table 1**. Overall, 60 women were enrolled, with 20 participants in each group. The data underscore marked differences in demographic, anthropometric, and endocrine. The HC and POI groups were of comparable young adult age and endocrine parameters (32.4 ± 4.7 vs. 32.2 ± 7.8 years, respectively), whereas the MP group was significantly older (56.9 ± 3.8 years). Importantly, the age at which ovarian insufficiency or menopause occurred was recorded for POI and MP participants. POI women experienced cessation of ovarian function markedly earlier than physiological timing (29.1 ± 7.4 years), confirming the “premature” nature of their condition. By contrast, MP women reported natural menopause at a mean age of 51.1 ± 2.5 years, which is consistent with population norms. Body mass index (BMI) followed a modest upward trend across the groups. HC women had the lowest BMI (21.4 ± 3.3 kg/m²), followed by POI women (22.5 ± 3.3 kg/m²) and then MP women (24.0 ± 1.6 kg/m²). Although these differences are numerically small, the gradual increase with age is consistent with known epidemiological patterns of weight gain through midlife. As for their characteristics of reproductive endocrinology, the most dramatic contrasts among the three groups appeared in the reproductive hormone panel, reflecting their divergent ovarian functional statuses. AMH is a widely accepted marker of ovarian reserve. HC women displayed robust concentrations (median 3.3 ng/mL with an interquartile range of 3.9), indicative of an ample follicular pool. In stark contrast, both POI and MP women had AMH levels below the assay’s functional detection limit (<0.02 ng/mL), confirming severe depletion or absence of growing follicles. Besides, FSH paralleled these findings. HC women had FSH concentrations in the normal premenopausal range (median 6.3 IU/L). POI women exhibited profoundly elevated FSH (median 74.8 IU/L), a biochemical hallmark of ovarian insufficiency resulting from loss of negative feedback from estradiol and inhibin B. MP women likewise showed high FSH (median 66.1 IU/L), although the median was slightly lower than that in POI. LH showed a similar pattern. HC women had low-normal LH (median 4.0 IU/L). POI women demonstrated marked LH elevation (median 44.1 IU/L), exceeding that observed in MP women (median 26.4 IU/L). E_2_ concentrations were highest in HC women (median 35.5 pg/mL), consistent with cyclic follicular activity. Both POI and MP women exhibited estradiol levels below the assay’s limit of quantification (<15 pg/mL), confirming hypoestrogenism. The undetectable E_2_ in POI is particularly striking given the woman’s young age, underscoring the severity of ovarian failure and its associated long-term health risks. Testosterone levels were not so different across all groups, with medians of 0.4 ng/mL in HC and POI women and 0.2 ng/mL in MP women.

**Table 1:** Baseline characteristics of all participant.

Characteristics	HC group	POI group	MP group
Number of cases	20	20	20
Age (years)	32.4 ± 4.7	32.2 ± 7.8	56.9 ± 3.8
Age at which POI or menopause occurs (years)	–	29.1 ± 7.4	51.1 ± 2.5
BMI (kg/m^2^)	21.4 ± 3.3	22.5 ± 3.3	24.0 ± 1.6
AMH (ng/mL)	3.3 (3.9)	<0.02*	<0.02*
FSH (IU/L)	6.3 (3.0)	74.8 (37.9)	66.1 (37.0)
LH (IU/L)	4.0 (1.9)	44.1 (36.6)	26.4 (17.3)
E2 (pg/mL)	35.5 (26.6)	<15.0*	<15.0*
T (ng/mL)	0.4 (0.2)	0.4 (0.4)	0.2 (0.2)

Mean (SD) for continuous variables or interquartile range (median).

*The average value is lower than the detection limit of the kit.

HC, healthy control; POI, premature ovarian insufficiency; MP, natural menopausal; BMI, body mass index; FSH, follicle-stimulating hormone; LH, luteinizing hormone; E2, estradiol; T, testosterone.

### 3.2 Proteomic profiles distinguish POI patient and healthy cohorts

Initially, PLS-DA revealed distinct clustering trends between the POI, MP and HC groups (**Figure 1A**). Permutation testing validated the reliability of the PLS-DA classification model. Subsequent pairwise comparative proteomic analysis screened differentially expressed proteins (DEPs) under the threshold of adjusted P (p.adj) < 0.05 and |log_2_FC|≥1. A total of 54 DEPs were identified between POI and HC groups, including 4 significantly upregulated and 50 downregulated proteins (**Figure 1B**). Meanwhile, 30 DEPs were detected when comparing POI with MP, among which 5 proteins were upregulated and 25 were downregulated (**Figure 1C**). Notably, both S100A8 and S100A9 displayed consistent significant upregulation in POI relative to both HC and MP groups. Furthermore, we utilized the DEPs to generate a heatmap, which clearly distinguished among the three groups (**Figures 1D, E**).

**Figure 1:**
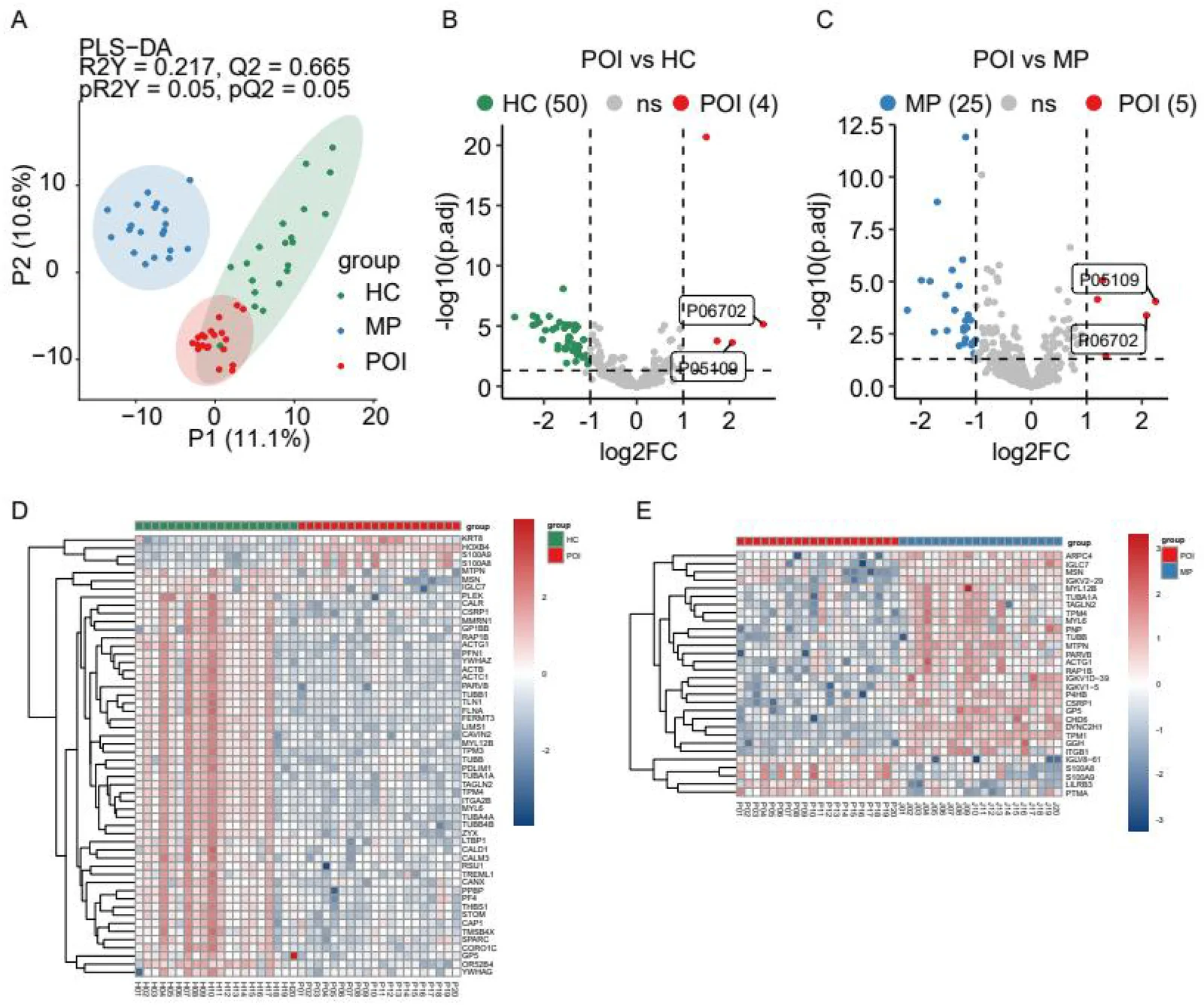
Proteomic analysis of DEPs among POI patients, MP and HC groups. **(A)** PLS-DA plot of proteomic data from each group. **(B)** Volcano plot of POI and HC proteins. **(C)** Volcano plot of POI and MP proteins. **(D)** Heatmap cluster of DEPs in the POI and HC groups. **(E)** Heatmap cluster of DEPs in the POI and MP groups.

To exclude the confounding effect of age differences between POI and MP cohorts, we conducted limma-based correlation analysis to evaluate the association between age and all serum proteins. After Benjamini–Hochberg FDR correction, no protein exhibited a significant age-related alteration (all adjusted P-values> 0.05, **Supplementary Table 2**), confirming that the intergroup proteomic disparities between POI and MP originate from POI pathogenesis rather than age-related physiological changes.

### 3.3 Functional enrichment analysis reveals dysregulation in cytoskeleton and inflammatory homeostasis in POI

Functional enrichment analysis of DEPs between POI and MP groups revealed significant pathway associations. For POI versus HC groups (**Figure 2A**), GO analysis showed dominant enrichment in wound healing, blood coagulation, actin cytoskeleton assembly and platelet activation, accompanied by inflammatory leukocyte aggregation and migration processes. Corresponding KEGG pathways were mainly enriched in motor protein, cell adhesion and leukocyte transendothelial migration signaling. For POI versus MP groups (**Figure 2B**), consistent enrichment of actin cytoskeleton and inflammatory leukocyte pathways was detected. Collectively, dysregulated cytoskeleton remodeling and leukocyte-associated inflammatory pathways were perturbed in POI relative to both HC and MP groups, representing shared functional signatures underlying POI pathogenesis.

**Figure 2:**
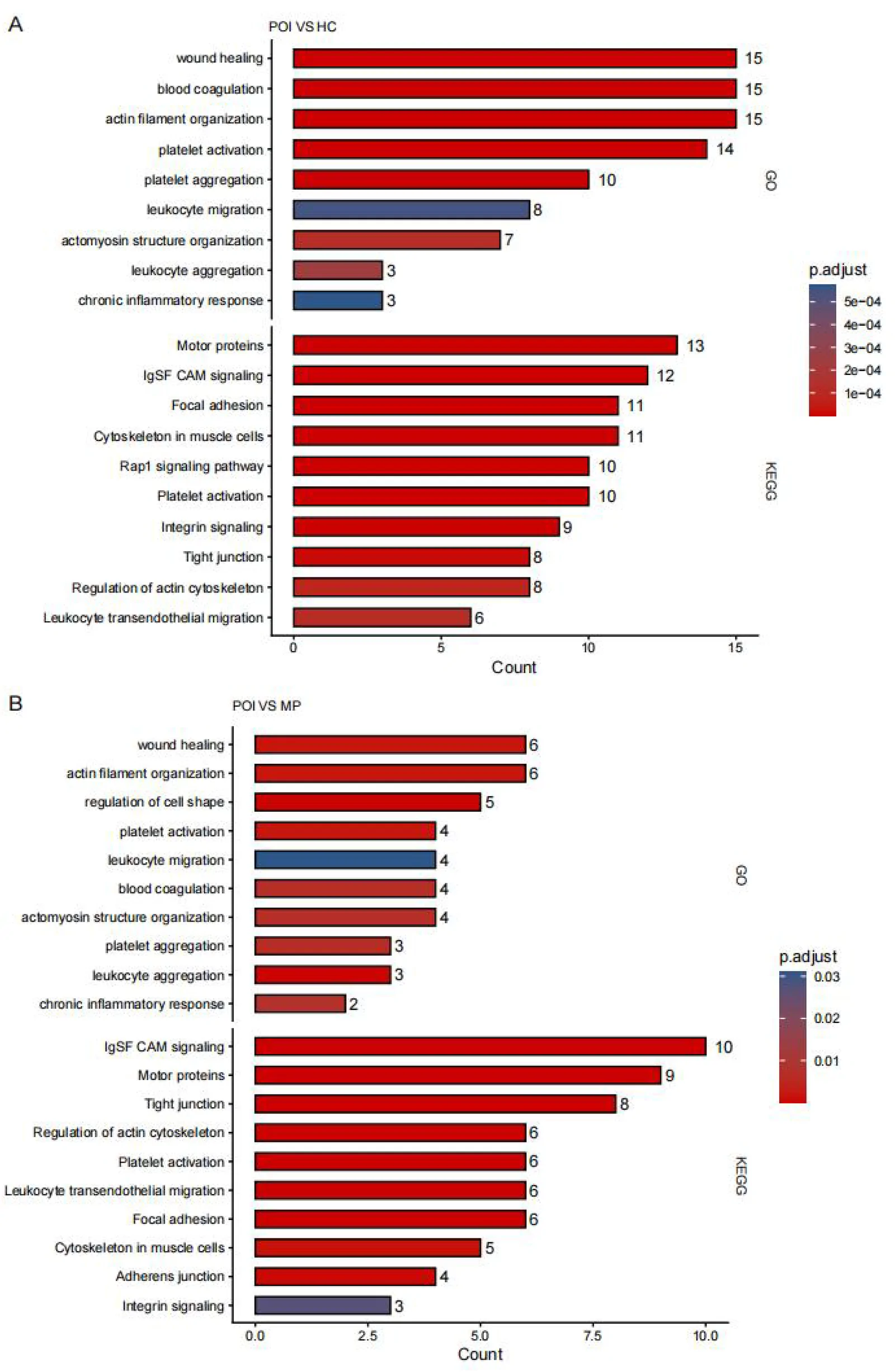
Protein function analysis among POI patients, MP and HC groups. **(A)** GO analysis and KEGG analysis of DEPs between POI and HC groups. **(B)** GO analysis and KEGG analysis of DEPs between POI and MP groups.

To quantify pathway activity across the three cohorts, we conducted Gene Set Variation Analysis (GSVA) using the MSigDB Hallmark gene set (50 canonical pathways). Three pathways (apical junction, epithelial-mesenchymal transition, and mitotic spindle) were significantly suppressed in POI relative to both HC and MP (**Supplementary Figure 1**). The uniformly reduced activity of these programs points to impaired cell adhesion, epithelial plasticity and mitotic stability, which together drive defective folliculogenesis and ovarian dysfunction in POI.

To further explore whether the core DEPs were involved in cytoskeleton and immune response, we constructed a protein-protein interaction (PPI) network comprising DEPs, which revealed significant associations and interaction strengths among these proteins. In the PPI network analysis of POI versus MP groups, cytoskeletal protein γ1-actin (ACTG1) functioned as the central hub node connecting multiple cytoskeletal proteins such as TPM1, TPM4 and MYL6. Immunoprotein S100A8 and S100A9 also demonstrated strong mutual binding (**Figure 3A**). Analysis of POI versus HC groups further revealed multiple cytoskeletal proteins had strong interactions, while β-actin (ACTB) and ACTG1 were located in a relatively core position (**Figure 3B**). Overall, these results demonstrated significant abnormalities in the cytoskeleton and inflammatory homeostasis in POI.

**Figure 3:**
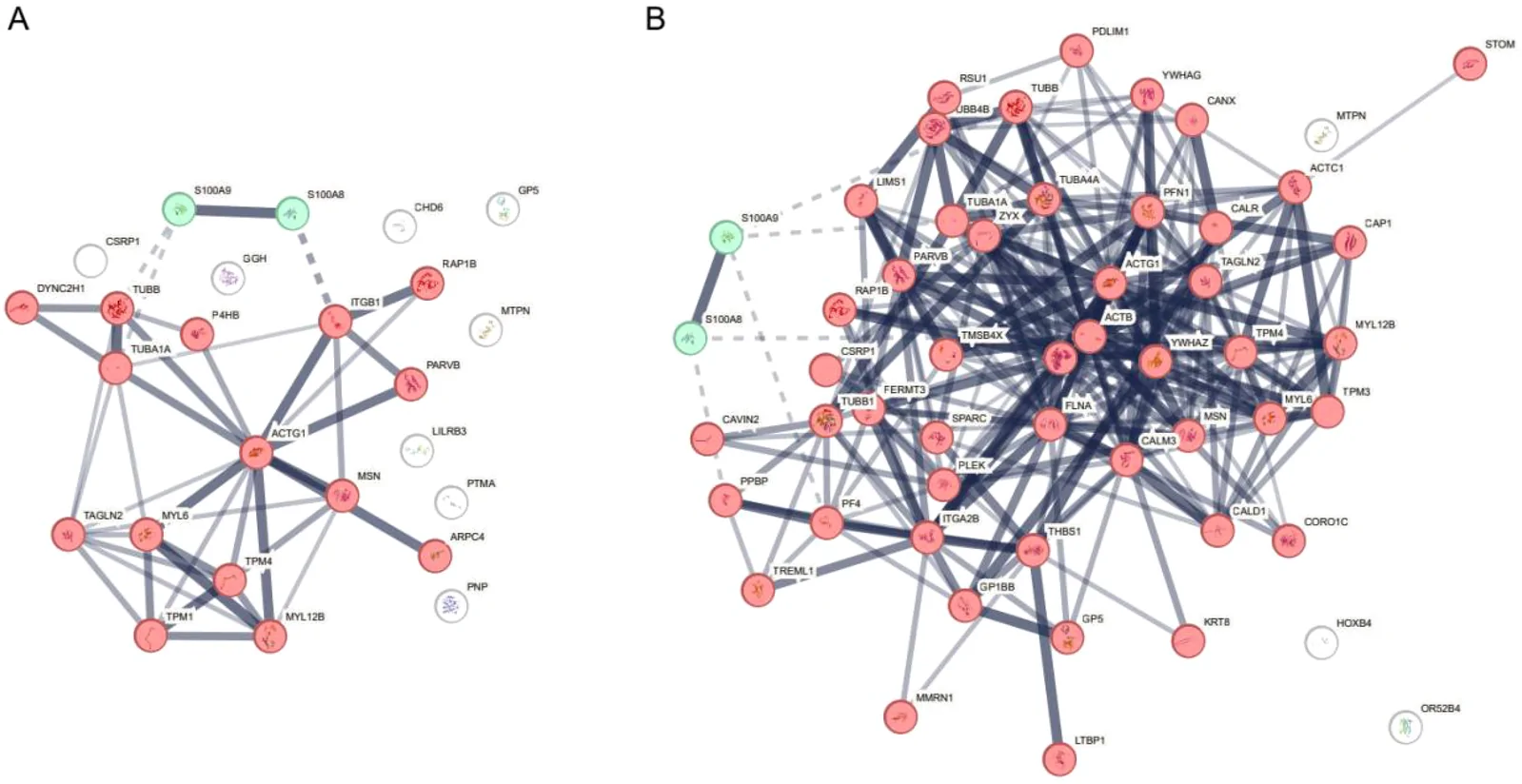
PPI network of DEPs among POI patients, MP and HC groups. Green indicates protein upregulation and red indicates downregulation. The thickness of the lines indicates the strength of the protein-protein interaction. **(A)** PPI network of DEPs between POI patients and MP groups. **(B)** PPI network of DEPs between POI patients and HC groups.

### 3.4 Identification and validation of POI biomarkers

To evaluate the diagnostic ability of biomarkers distinguishing between POI and MP groups, we further performed receiver operating characteristic (ROC) curve analyses on DEPs. Ten single candidate proteins, including CAVIN2, S100A8, S100A9, ITGA2B, LIMS1, MYL6, RAP1B, TPM4, OR52B4 and TUBA1A, all exhibited favorable discriminatory performance in the training set with AUC values ranging from 0.84 to 0.925. When validated against the MP cohort, each single protein maintained moderate-to-high predictive capacity, with validation AUCs between 0.693 and 0.845 (**Figures 4A–J**). Among these DEPs, five biomarkers were identified, including immune-related proteins S100A8 and S100A9, along with cytoskeleton regulatory proteins TUBA1A, TPM4 and RAP1B. All of these candidates achieved validation AUC values exceeding 0.8 and exhibited relatively small AUC attenuation (<11%), indicating reliable and stable diagnostic capacity for POI.

**Figure 4:**
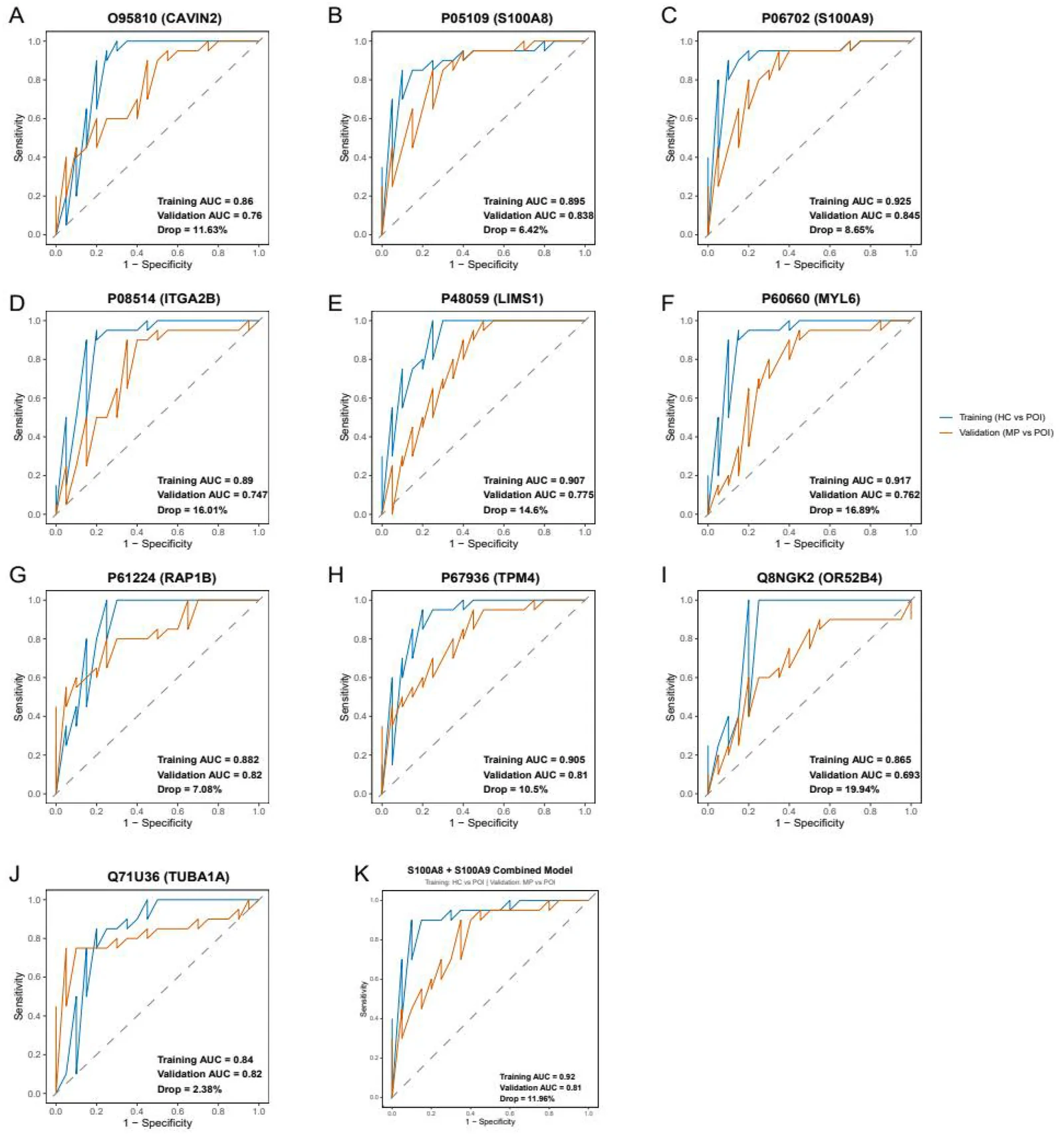
Identification of diagnostic biomarkers. Blue lines represent ROC curves derived from the training cohort (HC vs POI). Orange lines denote ROC curves validated in an independent cohort (MP vs POI). **(A–J)** Single-protein ROC analysis of CAVIN2, S100A8, S100A9, ITGA2B, LIMS1, MYL6, RAP1B, TPM4, OR52B4 and TUBA1A. **(K)** Combined diagnostic model based on S100A8/S100A9.

Notably, the inflammatory mediators S100A8 and S100A9 stood out from all candidates with superior and stable diagnostic performance. S100A8 and S100A9 both retained validation AUCs above 0.8 and showed relatively small performance attenuation when validated in the MP cohort. Moreover, the combined model of S100A8 and S100A9 achieved the best overall discriminatory power, with a training AUC of 0.92 and a validation AUC of 0.81 (**Figure 4K**). Collectively, immune-related proteins S100A8 and S100A9 are highly promising independent biomarkers for POI diagnosis, and their combination serves as an optimal diagnostic signature with reliable discriminatory ability to separate POI patients from both HC and MP populations.

To verify the robustness of these DEPs as potential biomarkers for POI, we performed pairwise correlation analyses between candidate protein abundances and key clinical indicators within each cohort. Within the POI patient cohort (**Figure 5A**), the inflammatory mediators S100A8 and S100A9 were significantly negatively correlated with AMH and positively correlated with FSH and LH. In contrast, cytoskeleton regulatory proteins RAP1B, TUBA1A and TPM4 exhibited positive correlations with AMH and negative correlations with FSH and LH. Within the MP cohort (**Figure 5B**), all five biomarkers showed significant correlations with chronological age and age at menopause, and opposing correlation patterns were observed between cytoskeleton regulatory proteins (TUBA1A, TPM4 and RAP1B) and S100A8/S100A9. Notably, only S100A8/S100A9 demonstrated significant correlations with serum estrogen and testosterone levels, whereas only TPM4 showed a weak negative correlation with LH. Collectively, these findings demonstrate that the five candidate biomarkers are tightly associated with clinical hormonal features of ovarian insufficiency and support their capacity to discriminate POI patients from healthy controls.

**Figure 5:**
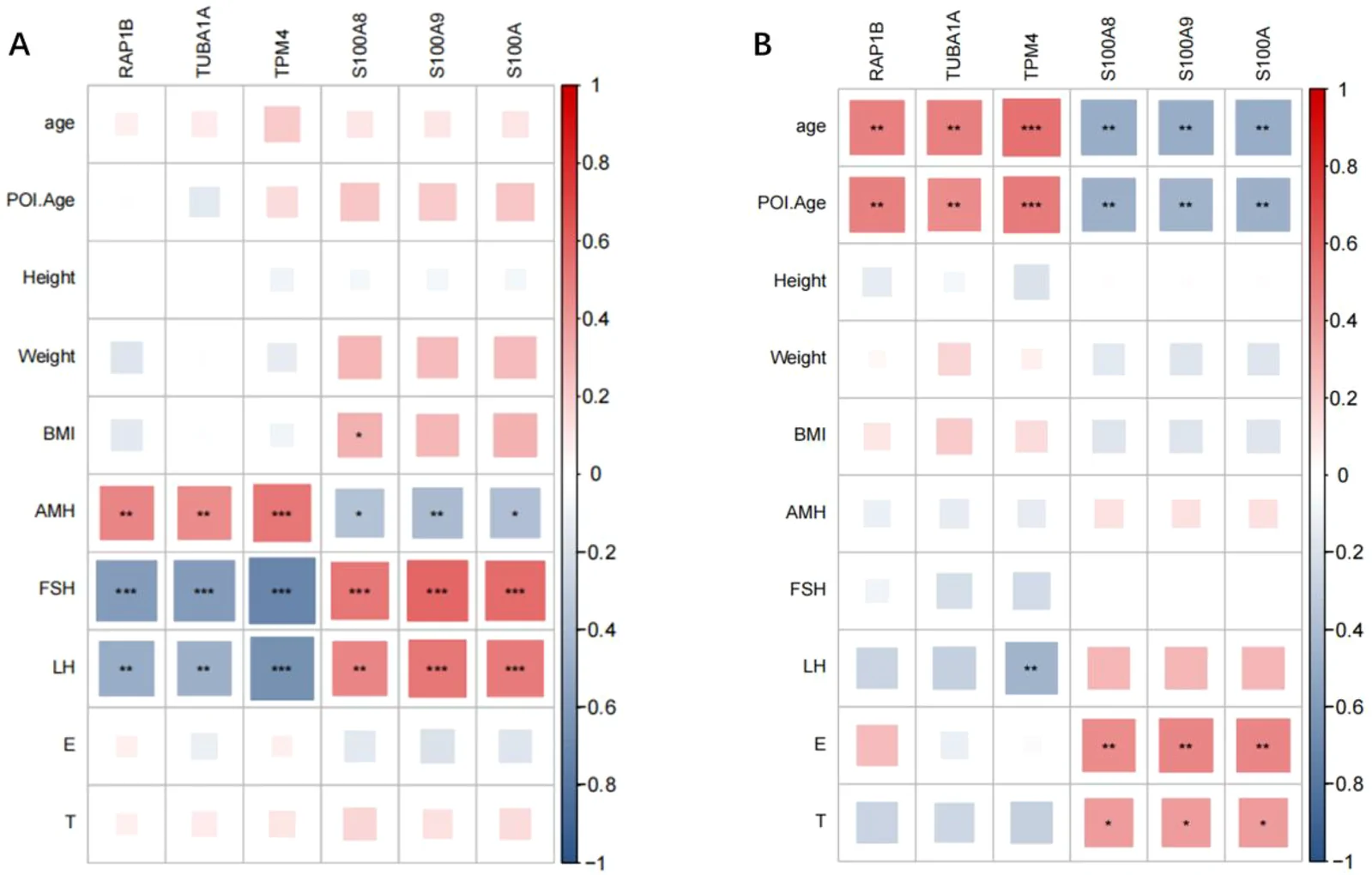
Correlations of candidate proteins with clinical indicators. **(A)** Correlation heatmap in the POI cohort. **(B)** Correlation heatmap in the MP cohort. Red indicates positive correlation, blue indicates negative correlation. POI.Age, age at POI diagnosis; LH, luteinizing hormone; FSH, follicle-stimulating hormone; T, testosterone; AMH, anti-Mullerian hormone; E, estrogen; BMI, body mass index; S100A, the combined S100A panel (S100A8+S100A9). *P < 0.05, **P < 0.01, ***P < 0.001.

To validate the potential of S100A8 and S100A9 as predictive biomarkers for POI, as suggested by prior proteomic analysis and their correlation with clinical features, we collected peripheral blood serum samples from a new cohort of 88 participants. This cohort included 44 patients with POI and 44 healthy controls. The results confirmed significantly elevated serum levels of S100A8 and S100A9 in POI patients compared to HC group (p<0.05). ROC curve analysis demonstrated that S100A8 and S100A9 exhibits a high diagnostic accuracy for distinguishing POI from HC (AUC = 0.849 and 0.861) (**Figures 6A, B**). The combined panel of S100A8 and S100A9 further improved discriminatory ability, with an AUC of 0.9 and a highly significant intergroup difference (p<0.05) between POI and HC groups, supporting their potential as biomarkers for POI (**Figure 6C**).

**Figure 6:**
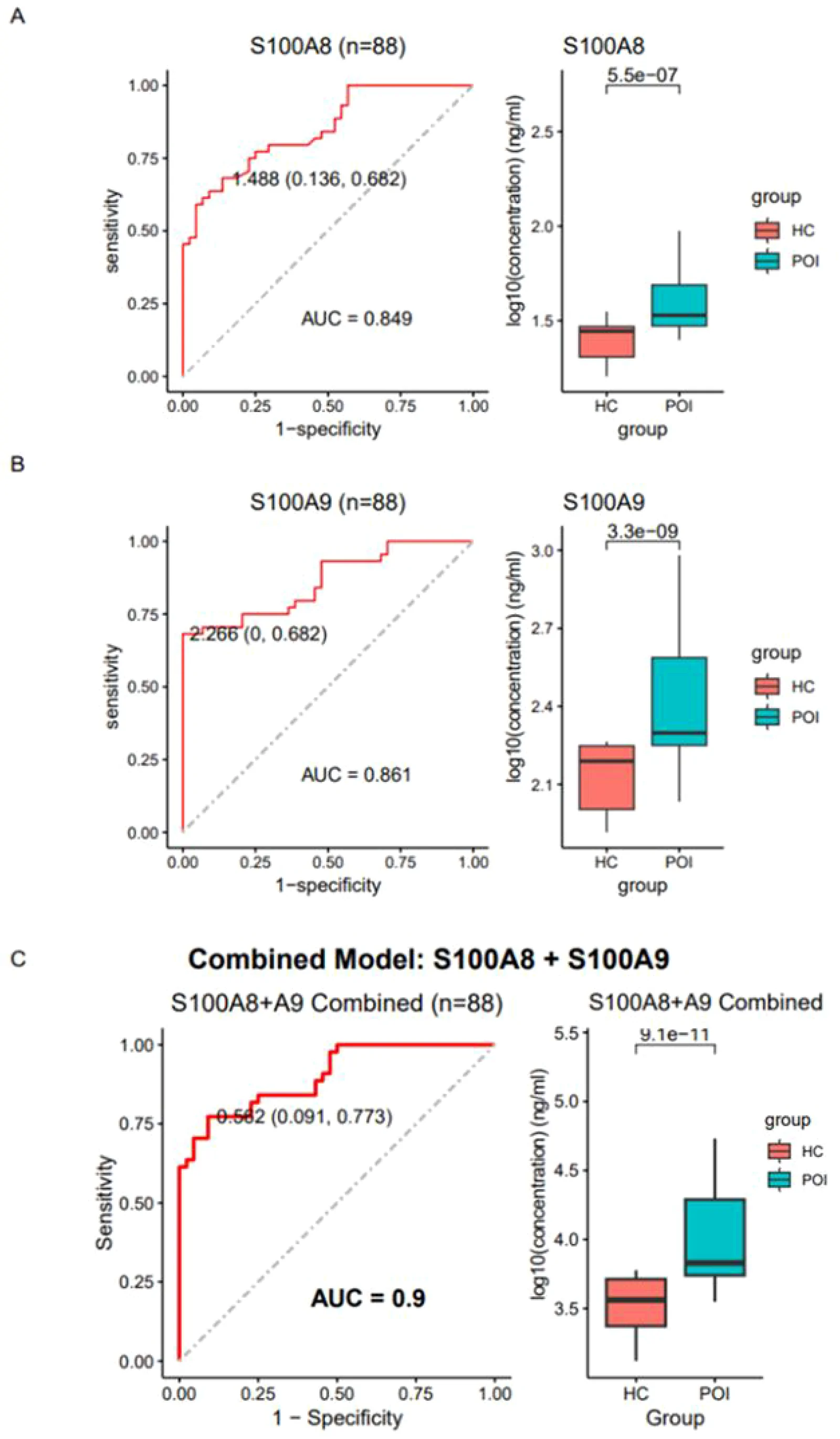
ELISA validation and diagnostic performance of serum S100A8 and S100A9 in the independent cohort (n=88). ROC curves (left) and group comparison boxplots (right) of **(A)** S100A8, **(B)** S100A9, and **(C)** S100A8+S100A9 combined model in HC and POI subjects.

## 4 Discussion

Through omics analysis of serum-derived proteins, we identified key disease pathways implicated in POI pathogenesis and discovered potential biomarkers for predicting POI onset. Comprehensive pathway enrichment analysis revealed significant dysregulation in cytoskeleton and inflammatory response pathways. The significant correlation between these selected differential proteins and previously reported clinical predictors supports their potential utility as candidate biomarkers, further suggesting significant abnormalities in cytoskeleton and inflammatory homeostasis in POI patients.

POI is a heterogeneous disease caused by a variety of mechanisms (1). However, in most cases, ovarian insufficiency occurs because of an anticipated depletion of the primordial follicular pool (9). The factors implicated in activating this mechanism exhibit significant heterogeneity, including genetic, autoimmune, metabolic, infectious, and iatrogenic origins (9, 10). Autoimmune ovarian inflammation has been widely implicated in the pathogenesis of POI. Our study demonstrated that S100A8 and S100A9 were significantly upregulated in patients with POI. S100A8 and S100A9, members of the S100 protein family, typically form a heterodimer to exert their biological functions. S100A8/S100A9 was widely expressed in many different cell types and were involved in the regulation of calcium homeostasis, glucose metabolism, cell proliferation, apoptosis, inflammation and tumorigenesis (11). Defined as damage-associated molecular patterns because of their pro-inflammatory properties, S100A8/S100A9 engaged Toll-like receptor 4 (TLR4) to activate downstream NF-κB and MAPK signaling pathways, thereby driving proinflammatory cytokine secretion and immune cell differentiation (11). Recently, single-cell sequencing of peripheral blood has also identified S100A9 as an immune-related candidate gene in POI, with a proposed model wherein S100A9 may induce the pyroptosis of granulosa cells and POI through TLR4 (8). In addition, serum concentrations of proinflammatory cytokines also showed that IL-17 and TNF-α were increased in POI patients (12). Another study has shown that Th17 cytokines (IL-17A and IL-17F) strongly induced expression of S100A9 and S100A8 in keratinocytes in patients with atopic dermatitis (13). These findings suggested that there was an interaction between Th17 cytokines and S100A8/9, both acting as proinflammatory factors to amplify the inflammatory response. However, S100A8/S100A9 was non-specific inflammatory markers rather than exclusive indicators of autoimmunity. Given the lack of autoantibody and complete immune cell profiling data in our retrospective cohort, we cannot confirm whether elevated S100A8/A9 levels originate from ovarian-specific autoimmune lesions or general systemic inflammation. The detailed mechanisms by which S100A8 and S100A9 contribute to POI pathogenesis require further validation.

In addition, our proteomic findings provide evidence of cytoskeletal abnormalities in POI patients. Previous studies have shown that cytoskeleton plays a critical role in ovarian follicle activation and degeneration (14–17). In our PPI network of POI, cytoskeleton-related proteins ACTB and ACTG1 are located in the core position. ACTB and ACTG1 constitute central components of the actin family, functioning as highly conserved cytoskeletal proteins that orchestrate cellular architecture, motility, and signal transduction (18). In ovarian tissues, ACTB has been identified and validated as a candidate biomarker for POI in humans (19). ACTG1 has been observed to significantly promote the migration and proliferation while inhibiting the apoptosis of granulosa cells in ovaries, suggesting that ACTG1 was required for ovarian remodeling (20). In addition, our research further observed that multiple cytoskeleton-related proteins including TPM1, TPM4, TUBA1A, and RAP1B, were all down-regulated to varying degrees in POI. Consequently, the proteomic profile indicates that dysregulation of cytoskeleton organization likely contributes to POI pathogenesis. Based on the POI-related inflammatory biomarkers identified in this work, novel therapeutic strategies centered on food-medicine homology, gut microbiota modulation and multi-omics screening provide innovative approaches to intervene ovarian inflammatory damage (21–24).

Notably, conventional endocrine markers (FSH, AMH) only show obvious abnormalities when ovarian follicle reserve has already suffered depletion, which limits their value for early warning of subclinical POI. For women at the pre-failure stage with normal-range FSH and slightly reduced AMH, routine hormone detection cannot stratify individuals at high risk of progressive ovarian dysfunction. In contrast, serum S100A8/S100A9 reflect early systemic inflammatory perturbations associated with ovarian damage, and they can serve as auxiliary biomarkers to screen subclinical POI patients before prominent hormonal disorders occur, providing supplementary diagnostic value beyond routine hormonal workup.

This study has several limitations. First, the sample size is relatively small. Owing to inter-individual heterogeneity, the cohort may lack adequate statistical power, and our findings require validation in larger populations. Second, although age-adjusted analysis was used to reduce confounding, residual age-related interference cannot be fully excluded. Third, retrospectively archived samples lack complete clinical data, including blood cell counts, autoantibody status, disease course and medication information. Therefore, we cannot distinguish ovarian local inflammation from systemic inflammation when interpreting changes in S100A8/A9 and cytoskeletal proteins, and we cannot attribute elevated S100A8/A9 to autoimmune abnormalities specifically. Fourth, hemolysis testing and high-abundance protein depletion were not conducted for proteomic detection, though peptide length filtering was adopted to mitigate exogenous contamination. Of note, AMH, FSH, and LH were not recovered among the differentially expressed proteins, presumably due to their very low serum concentrations and heavy glycosylation, which may impair tryptic peptide generation and compromise MS-based identification in label-free serum proteomics. Finally, limited by experimental resources, only S100A8 and S100A9 were verified by ELISA, and other candidate proteins await further validation. Prospective studies with comprehensive clinical and immune data are needed to confirm our results.

This study employed proteomic profiling of peripheral blood serum from women with POI, healthy controls, and menopausal individuals to identify potential diagnostic biomarkers for POI. The proteomic profile indicated that numerous DEPs effectively distinguish the POI group from other groups with statistical significance. Comprehensive pathway enrichment analysis revealed significant dysregulation in cytoskeleton organization and inflammatory signaling in POI. Furthermore, we identified a panel of five candidate proteins (S100A8, S100A9, TUBA1A, TPM4, and RAP1B) that demonstrated robust potential for POI prediction. Specifically, the consistent validation of S100A8/S100A9 via ELISA reinforces the reliability of our proteomic screening, highlighting their particular clinical utility as biomarkers for POI.

## Data Availability

The datasets presented in this study can be found in online repositories. The names of the repository/repositories and accession number(s) can be found in the article/**Supplementary Material**.
