# Supplementary material for: Proteomics reveal serum biomarkers for early diagnosis in premature ovarian insufficiency
Source: Front Endocrinol (Lausanne). 2026 Sep 17;17:1917911. doi: 10.3389/fendo.2026.1917911 (PMC13626912; doi:10.3389/fendo.2026.1917911)
Supplement: Supplementary file 1 [file DataSheet1.pdf]

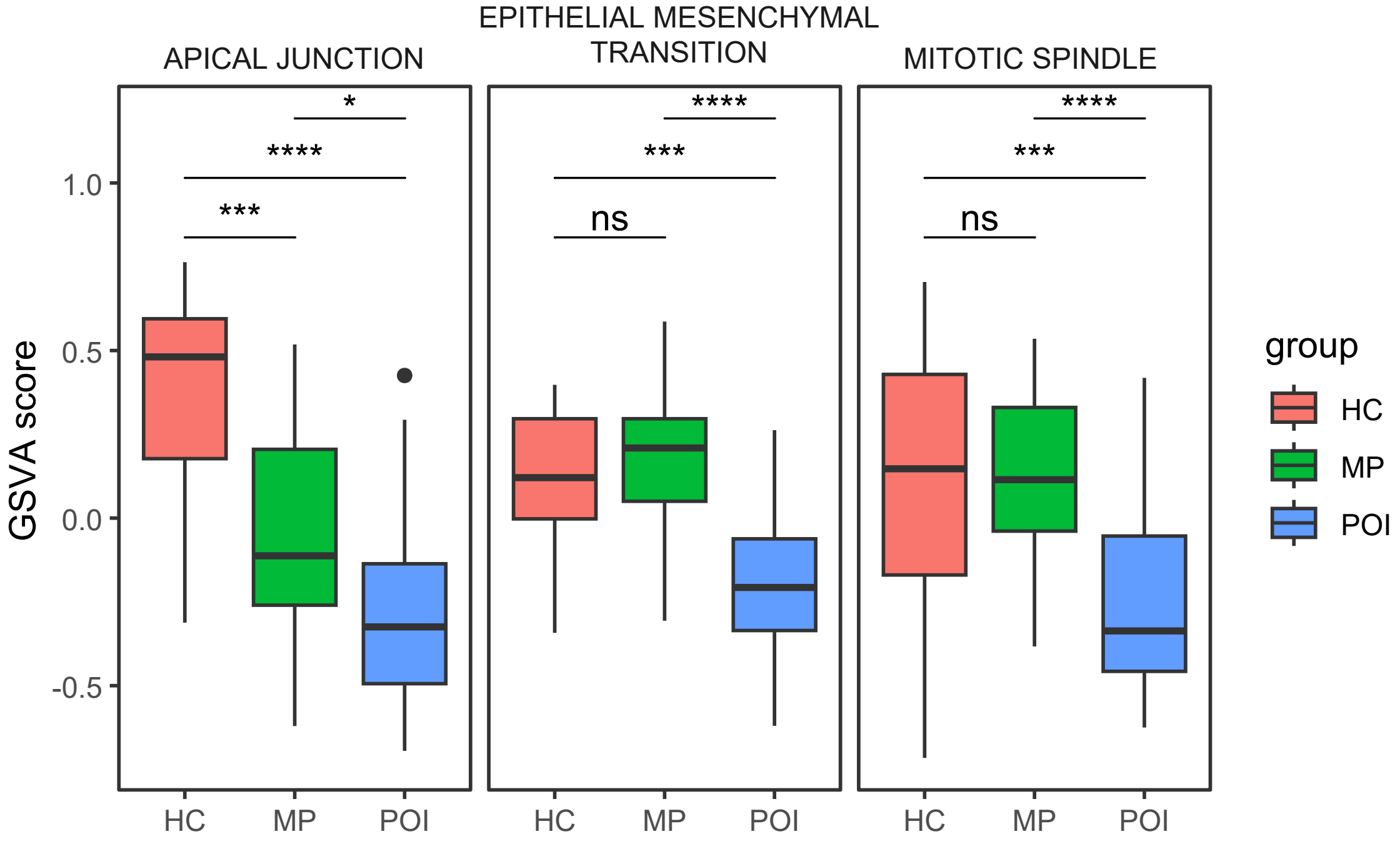

APICAL JUNCTION
EPITHELIAL MESENCHYMAL TRANSITION
MITOTIC SPINDLE
GSVA score
1.0
0.5
0.0
-0.5
*
****
***
****
***
ns
****
***
ns
HC
MP
POI
group
HC
MP
POI
